# Evaluation of Essential and Toxic Elements in Amniotic Fluid and Maternal Serum at Birth

**DOI:** 10.1007/s12011-018-1471-2

**Published:** 2018-08-10

**Authors:** Rafał Kocyłowski, Mariusz Grzesiak, Zuzanna Gaj, Wiktor Lorenc, Ewa Bakinowska, Danuta Barałkiewicz, Constantin Sylvius von Kaisenberg, Joanna Suliburska

**Affiliations:** 1 Department of Obstretrics, Perinatology and Gynecology, Polish Mother’s Memorial Hospital-Research Institute, ul. Rzgowska 281/289, 93-338 Łódz, Poland; 2 PreMediCare New Med Medical Centre, ul. Drużbickiego 13, 61-693 Poznań, Poland; 3 Scientific Laboratory of the Center of Medical Laboratory Diagnostics and Screening, Polish Mother’s Memorial Hospital-Research Institute, ul. Rzgowska 281/289, 93-338 Łódz, Poland; 4 Department of Trace Element Analysis by Spectroscopy Method, Faculty of Chemistry, Adam Mickiewicz University in Poznań, ul. Umultowska 89b, 61-614 Poznan, Poland; 5 Institute of Mathematics, Poznan University of Technology, 5 M. Skłodowska-Curie Square, 60-965 Poznan, Poland; 6 Department of Obstetrics and Gynecology, Hannover Medical School, Carl-Neuberg-Str. 1, 30625 Hannover, Germany; 7 Institute of Human Nutrition and Dietetics, Poznan University of Life Sciences, ul. Wojska Polskiego 31, 60-624 Poznań, Poland

**Keywords:** Essential and toxic elements, Amniotic fluid, Maternal serum, Delivery, Reference values

## Abstract

The objective of this study was to determine the concentration and the reference ranges of essential and toxic elements in amniotic fluid (AF) and maternal serum (MS) at birth. This study was conducted among 175 healthy pregnant Caucasian European women aged 18–42. AF and maternal blood samples were collected during delivery. An inductively coupled plasma mass spectrometry (ICP-MS) technique was used to determine the levels of Mg, Co, Cu, Zn, Sr, Cd, Ba, Pb, U, Ca, Cr, Al, Mn, V, Fe, As, Se and Sb in AF and MS. The range of reference values was calculated for all analyzed elements in the serum and AF. The mean concentrations of elements, except Pb, were generally higher in MS than in AF. Multiple regression analysis showed that the maternal/newborn body mass (MBM/NBM) ratio was a strong negative predictor (among maternal age and gravidity) of Mg concentration in amniotic fluid. In the serum, MBN/NBM ratio was a strong positive predictor of Cu concentration. Moreover, regression analysis showed that maternal age was an independent positive predictor of the Se level in maternal serum. The reference value ranges of 18 essential and toxic elements were established in AF and MS among a population of healthy pregnant Polish women at delivery. The level of Mg, Co, Cu, Ca and Se in AF and MS can be determined by maternal age and MBM/NBM ratio. These results can be useful in counseling individuals with pregnancies affected by exposure to one of the parameters under investigation.

**Electronic supplementary material:**

The online version of this article (10.1007/s12011-018-1471-2) contains supplementary material, which is available to authorized users.

## Introduction

The composition of amniotic fluid (AF) is important for fetal development and reflects both maternal and fetal compartments. The content of elements in the AF can provide important information on the nutritional and mineral status of the mother and her fetus as well as their exposure to toxic elements [1–3]. During pregnancy, physiologic and metabolic changes in the woman’s organs depend on the requirements of the growing fetus. The demand for some trace elements increases during pregnancy [1]. Therefore, a deficiency in some minerals is often observed among pregnant women [1, 4, 5]. Inadequate quantities of micro- and macro-minerals as well as overexposure to toxic elements could be detrimental to the health of both the pregnant woman and the fetus. An evaluation of the concentration of trace elements in maternal blood and amniotic fluid was performed in different countries and populations [6–11]. However, differences between these data occur that are mainly caused by differences in regions (e.g. Europe vs Asia), environmental factors and racial differences as well as week of gestation, eating behavior and health status of the women.

Literature on this subject does not specify updated reference values for concentrations of elements in the serum and AF of healthy pregnant Polish women. Preliminary research data and that in the literature available on the subject show that there are dependencies between the concentrations of certain elements—especially magnesium, copper, cadmium and barium—and gestational age and fetal biometric specifications [6]. Moreover, there is a lack of thorough exploration, including that pertaining to macro-minerals, micro-minerals and toxic elements, in maternal and fetal compartments in the course of physiological and pathological pregnancies by using modern standard analytical methods with inductively coupled plasma (ICP-MS) technique, which has become the accepted standard in recent years [12].

New knowledge about concentrations of elements in the AF and maternal serum (MS) in the course of gestation will improve our understanding of the physiological and pathological processes during pregnancy, might help to develop novel methods for prenatal diagnosis and possibly contribute to the prevention of some pregnancy-related complications. Therefore, the main aim of the research is to determine the reference values for concentrations of essential and toxic elements in the MS and AF of women at the time of delivery.

## Material and Methods

### Study Patients

The study protocol was approved by the Local Ethics Committee of the Polish Mother’s Memorial Hospital Research Institute in Łódź, Poland (approval no. 50/2016). This study was conducted in accordance with the Declaration of Helsinki.

The study group consisted of a total of 175 examined term deliveries of healthy European women (average, 39 weeks of gestation; range, 38–42 weeks), by vaginal birth (73%) or by caesarean section (27%) of singleton pregnancies without birth defects. The study included 103 (59%) male and 72 (41%) female babies. The median maternal age was 29.5 ± 4.6 years (range, 18–42 years). The AF and maternal blood were collected concurrently at birth.

The *inclusion criteria* stated that the pregnancy should be a single intrauterine gestation without apparent congenital anomalies on evaluation by ultrasound or prenatal tests, without any signs and symptoms of maternal or fetal infection or anemia and that the patient should give informed consent.

The *exclusion criteria* included abnormal vaginal bleeding, placental hematoma, premature rupture of membranes (PROM), placental insufficiency, multiple pregnancy, maternal diabetes, hypertension in pregnancy, obstetric history with multiple miscarriages and fetal growth disorders (e.g. growth restriction, macrosomia), birth defects, or genetic complications in the pregnancy or in the women, current use of drugs or supplements that affect the mineral balance in the organism and maternal exposure to alcohol, cocaine or tobacco smoke.

### Study Design

All subjects were informed of the study’s aims, procedures and measurement methods, and the written consent of each patient for study participation was obtained. The maternal weight was measured before delivery to the nearest 0.1 kg using a calibrated digital weighing scale with the subjects in lightweight clothing. The body mass index (BMI) was calculated after obtaining the weight and height. The newborns’ weight was measured after birth using a digital weighing scale when naked. The week of gestation was determined on the basis of the first trimester prenatal ultrasonography.

Blood samples were collected from a maternal vein at the time of delivery. Monovette test tubes (neutral or serum Z/7.5 mL; Sarstedt, Sarstedt AG & Co, Nümbrecht, Germany) were used with and without anticoagulants to obtain whole blood and blood serum. Serum samples were frozen and stored at − 80 °C. All hematologic specifications (red blood cell count, hemoglobin and hematocrit) were evaluated using a Sysmex XN-2000 automated cell counter (The Sysmex Company, Kobe, Japan).

The AF samples were obtained (5 mL) by using either a transabdominal puncture in the surgical wound with intact membranes using sterile needles and syringes during cesarean delivery or with sterile needles and syringes to puncture the amniotic sac while using a vaginal speculum to visualize intact membranes when cervical dilatation was 4 cm or more during vaginal birth. The samples were centrifuged (3000 rpm/min for 10 min at 4 °C), frozen and stored at − 80 °C.

Blood loss during delivery was determined by a semi-quantitative method. The placentas were collected and evaluated immediately after delivery. The weight of the placenta with its attached membranes and umbilical cord was determined by using an electronic baby weighing scale.

### Mineral Analysis and Sample Preparation

Samples of AF and MS were frozen directly after collection in sealed polypropylene tubes until further treatment. Samples were thawed at room temperature and mineralized in a high-pressure, closed, microwave digestion system (Ethos One, Milestone). Thereafter, 1 mL of the sample was transferred to quartz vessels with 0.5 mL of 65% HNO_3_ (Suprapur, Merck) and 0.5 mL of 30% H_2_O_2_ (Traceselect, Sigma Aldrich) and placed in a sealed PTFE container. The digestion program was conducted in three steps: (1) 20 min of ramp time until 180 °C with maximum power of 1500 W, (2) 40 min of hold time at 180 °C and maximum power of 1500 W and (3) 30 min of cooling time. The solutions thus obtained were quantitatively transferred to the volumetric flask and diluted to 10 mL with demineralized water (TKA Smart2Pure, Niederelbert, Germany). All laboratory equipment and containers were thoroughly rinsed with 1% HNO_3_ and demineralized water. The procedural blank solutions were prepared in the same manner as the tested samples.

### Analytical Procedure

An Elan DRC II ICP-MS (PerkinElmer SCIEX, Ontario, Canada) was used to determine Mg, Co, Cu, Zn, Sr, Cd, Ba, Pb, U, Ca, Cr, Al, Mn, V, Fe, As, Se and Sb concentrations. The sample was introduced into argon (Linde Gas, Poland) plasma via a cyclonic spray chamber, a concentric glass nebulizer and a quartz torch with a quartz injector. The operating conditions for the inductively coupled plasma mass spectrometry (ICP-MS) were optimized on a daily basis and were as follows: RF power was 1050–1150 W; the plasma gas flow rate was 16 L min^−1^; the nebulizer gas flow rates was 0.89–0.91 L min^−1^ and the auxiliary gas flow rate was 1.2 L min^−1^. The dynamic reaction cell (DRC) mode was used in order to eliminate spectral interferences with high-purity ammonia (Linde Gas, Poland) and high-purity oxygen (Linde Gas, Poland) as DRC reaction gases.^27^Al, ^44^Ca, ^51^V, ^52^Cr and ^55^Mn were analyzed in DRC mode with ammonia and ^78^Se, ^91^AsO and ^121^Sb with oxygen as the DRC gas. ^75^As^+^ in the presence of oxygen in DRC readily forms a thermodynamically stable ion ^91^AsO^+^ with a high level of efficiency, which is free of any Ar-matrix interferences. To eliminate non-spectral interferences, a 10 μg L^−1^ solution of ^45^Sc, ^74^Ge, ^103^Rh and ^159^Tb was used as an internal standard. A multi-element stock solution (Multi-element Calibration Standard 3, Atomic Spectroscopy Standard, PerkinElmer Pure) containing the analyzed elements at a concentration of 10 mg L^−1^ and single-element 1000 mg L^−1^ Sb solution (Certipur, Merck) were used to prepare a series of standard solutions for calibration. A calibration based on a weighted least squares calibration curve was employed for all elements. The ranges of the calibration curves were 5–1500 μg L^−1^ for ^24^Mg and ^44^Ca, 5–1000 μg L^−1^ for ^57^Fe, 0.5–200 μg L^−1^ for ^27^Al and ^66^Zn and 0.05–20 μg L^−1^ for ^51^V, ^52^Cr, ^55^Mn, ^59^Co, ^63^Cu, ^75^As, ^78^Se, ^111^Cd, ^121^Sb, ^138^Ba, ^208^Pb and ^238^U. The linearity—calculated as *R*^2^—was acceptable for all analyzed elements (*R*^2^ > 0.999). The trueness of the analytical method was assessed by analyzing the certified reference material (CRM) Seronorm™ Trace Elements Serum L-2 which was subjected to the preparation steps according to the above-described procedure. Table 1 contains a comparison of the certified values of the applied CRM and measured the values with respective uncertainties and the calculated recovery of the certified values. The values of recovery are within an acceptable range for all analytes, which demonstrates that the described analytical procedure is fit for the intended purpose.

**Table 1:** Comparison of certified values of applied CRM and measured values

	Certified value (μg L^−1^)	Measured value (μg L^−1^)	Recovery (%)
Mg	40,800 ± 4700	38,100 ± 7200	93
Al	104 ± 6	118 ± 17	113
Ca	145,000 ± 8000	138,000 ± 2100	95
V	1.01*	1.082 ± 0.091	107
Cr	4.8 ± 0.4	4.23 ± 0.80	88
Mn	19.9 ± 1.1	18.6 ± 3.4	93
Co	3.2 ± 0.2	3.09 ± 0.68	97
Cu	2887 ± 99	2574 ± 309	89
Zn	2520 ± 206	2218 ± 510	88
Fe	2030 ± 130	2331 ± 225	115
As	0.67*	0.648 ± 0.068	97
Se	163 ± 10	194 ± 31	119
Sr	36.3*	40.0 ± 1.8	110
Cd	0.13*	0.110 ± 0.011	85
Sb	80*	83.2 ± 3.0	104
Ba	139*	154.0 ± 9.3	111
Pb	1.11*	1.39 ± 0.12	125
U	0.048*	0.0522 ± 0.0016	109

*Approximate values

### Statistical Analysis

A detailed statistical analysis was conducted with the RStudio software (R version 3.4.0) R Core Team (2017). Basic sample statistics, such as measures of location, minimum, maximum, median, mean and standard deviation, were conducted. Moreover, the confidence interval was set at 95% for the unknown expected value of the content of the elements. All original data were illustrated in box-plots. In addition, paired comparison analysis was conducted (both in the AF and the MS). To apply the adequate test at the first stage, the normality of all variables was checked by means of a Shapiro–Wilk normality test.

Based on the results of the Shapiro–Wilk normality test, to conduct the paired comparison analysis, a *t* test was chosen to compare two population means: the average content of Mg in AF with the average content of Mg in the MS. The subsequent analyses were based on the Wilcoxon’s test. The testing was set at the statistical significance level of *p* = 0.05. The concentration of the elements was presented in the form of box-plots.

To evaluate whether the chosen factors significantly influence the content of individual elements in the fluid and the serum, an analysis of multiple regression was introduced. At the first stage, an analysis was conducted to determine which of the 18 elements, both in the AF and the MS, can be tested in terms of the significance of the multiple regression coefficient. The normality of the residuals from the model was checked by using a quantile-quantile chart (Q-Q). The *x*-axis represents theoretical quantiles (as the inverse cumulative distribution function for normal variables), and the *y*-axis shows quantiles from the sample for the model residuals. If the distribution of these points is linear, it may be assumed that the residuals form a normal distribution. This assumption was confirmed by using the Shapiro–Wilk normality test. The normality of the residuals was confirmed for four elements in the AF (Mg_AF, Co_AF, Cu_AF and Ca_AF) and for five elements in the MS (Mg_S, Co_S, Cu_S, Cd_S and Se_S). The significance of the factors was analyzed at the levels: *p = 0.1, **p = 0.05 and ***p = 0.01.

## Results

**Fig. 1:**
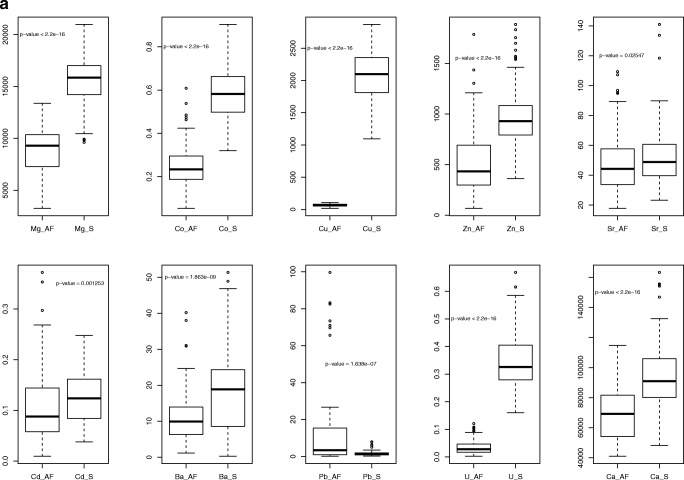

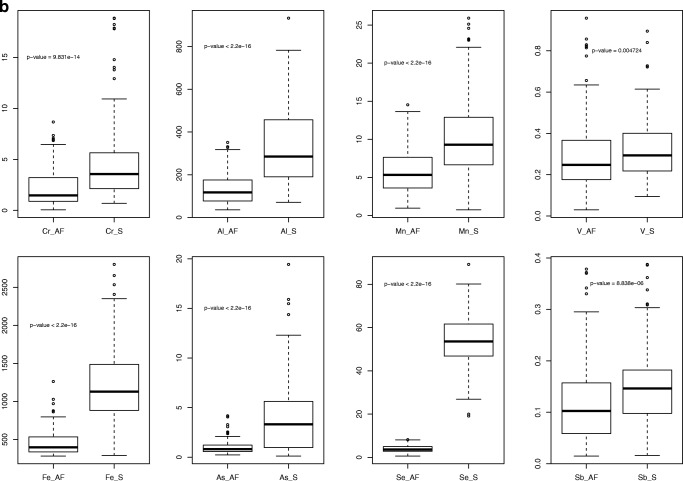
**a**, **b** The concentration of elements in amniotic fluid and maternal serum

Maternal and newborn’s parameters are shown in Table 2. The concentration of the elements (except for Pb) was significantly higher in the MS than in the AF (Fig. 1). The Pb level was markedly lower in the MS than in the AF (Fig. 1). The reference values of the 18 elements in the AF and MS for pregnant women at delivery are listed in Tables 3 and 4.

**Table 2:** Characteristic of women (mean ± SD/median/min-max)

Maternal parameters		Newborn’s parameters	
Parameter	Value	Parameter	Value
Number of women	175	Gender F/M	103/72
Age of mother (years)	29.5 ± 4.6 30 18–42	Birth weight (g)	3367 ± 405.9 3350 2350–5000
Weight of mother in pregnancy (kg)	76.8 ± 11.8 75 53–124	Apgar score	9.5 ± 0.6 10 7–10
BMI of mother before pregnancy (kg/m^2^)	22.3 ± 3.7 21.5 16.3–37.8		
Week of pregnancy	39.1 ± 1.2 39 38–42		
Gravidity			
0	51%		
1	28%		
2	12%		
3	4%		
≥ 4	5%		
Blood loss (mL)	320 ± 78.2 300 150–500		
Weight of placenta	538.7 ± 105.8 520 320–1020		
Hb (g/dL)	12.3 ± 1.0 12.3 8.7–14.6		
Hct (%)	36.3 ± 2.7 36.1 29.3–42.1		
RBC (10^12^/L)	4.2 ± 0.3 4.2 3.3–5.0		

*SD* standard deviation, *min* minimum, *max* maximum, *Hb* hemoglobin, *Hct* hematocrit, *RBC* red blood cells, *y* years, *F* female, *M* male

**Table 3:** Elements concentration in maternal serum (μg L^−1^)

	Min	Max	Median	Mean	SD	Reference values range (2.5th–97.5th percentile)
Mg	9617	20,976	15,849	15,567	2377	15,189–15,945
Co	0.320	0.903	0.582	0.593	0.135	0.570–0.615
Cu	1094	2869	2097	2103	366.8	2043–2163
Zn	361.5	1885	929.0	971.2	278.8	927.0–1015
Sr	23.2	141.0	48.81	52.23	20.01	48.54–55.92
Cd	0.038	0.248	0.124	0.126	0.051	0.116–0.137
Ba	0.278	51.35	18.87	18.12	11.24	16.32–19.92
Pb	0.203	7.93	1.30	1.62	1.29	1.40–1.85
U	0.160	0.669	0.326	0.348	0.100	0.333–0.364
Ca	48,240	163,350	90,952	93,976	21,014	90,668–97,285
Cr	0.698	18.86	3.56	4.77	4.03	4.06–5.47
Al.	71.22	933.0	284.8	334.8	181.9	305.7–363.9
Mn	0.749	25.91	9.30	10.54	5.33	9.63–11.44
V	0.095	0.895	0.293	0.321	0.140	0.299–0.343
Fe	291.7	2802	1130	1245	507.9	1156–1333
As	0.118	19.43	3.33	3.85	3.39	3.30–4.40
Se	19.09	89.19	53.54	54.41	12.10	52.47–56.35
Sb	0.016	0.387	0.146	0.152	0.073	0.140–0.164

*Min* minimum value, *max* maximum value, *sd* standard deviation

**Table 4:** Elements concentration in amniotic fluid (ug L^−1^)

	Min	Max	Median	Mean	Sd	Reference values range (2.5th–97.5th percentile)
Mg	3267	13,377	9297	9004	2085	8686–9321
Co	0.053	0.608	0.233	0.243	0.094	0.229–0.258
Cu	17.88	108.8	64.92	67.73	18.56	64.84–70.61
Zn	67.73	1787	433.5	506.3	285.1	463.5–549.2
Sr	17.84	109.42	44.28	47.64	19.17	44.48–50.81
Cd	0.009	0.372	0.088	0.109	0.071	0.097–0.121
Ba	1.15	40.20	9.93	10.93	6.60	9.94–11.92
Pb	0.088	99.70	3.36	10.23	17.32	7.43–13.03
U	0.002	0.121	0.028	0.036	0.0245	0.032–0.040
Ca	41,003	114,733	69,264	69,925	17,461	67,228–72,623
Cr	0.057	8.67	1.48	2.17	1.84	1.87–2.47
Al.	35.76	351.17	117.68	131.04	66.46	120.9–141.2
Mn	0.977	14.53	5.34	5.85	2.83	5.41–6.29
V	0.030	0.959	0.248	0.293	0.170	0.268–0.319
Fe	282.7	1263.1	398.3	466.0	180.1	433.1–498.9
As	0.237	4.174	0.823	1.02	0.703	0.906–1.14
Se	0.579	8.16	3.65	3.93	1.59	3.67–4.18
Sb	0.015	0.378	0.103	0.118	0.076	0.106–0.130

*Min* minimum value, *max* maximum value, *sd* standard deviation

Several factors such as maternal age, maternal body mass, newborn body mass, the MBM/NBM ratio, gravidity, parity and gender were assessed to determine the concentration of elements in AF and MS using regression analysis.

Before the multiple regression analysis was conducted, we determined which of the 18 elements in the AF and MS were appropriate for testing the significance of the multiple regression coefficient. The normality of the residuals was confirmed for four elements in the AF (Mg, Co, Cu and Ca) and five elements in the MS (Mg, Co, Cu, Cd and Se) (see Supplementary Material).

The results of multiple regression analysis showed that the maternal body mass/newborn body mass (MBM/NBM) ratio was a strong negative predictor (among maternal age and gravidity) of Mg concentration in the AF (Tables 5). In maternal serum, MBN/NBM was a strong and positive predictor of Cu concentration (Table 6). Furthermore, regression analysis showed that maternal age was a strong positive predictor of Se level in the maternal serum (Table 6).

**Table 5:** The predictors of the multiple regression of minerals concentration in amniotic fluid (β-coefficient)

Amniotic fluid	Mg	Co	Cu	Ca
Maternal age	0.023	0.0013	0.22	− 175.8
Maternal/newborn body weight	− 113.92**	0.00026	− 0.064	− 227.3
Parity	127.05	− 0.0069	1.09	975.5

*p = 0.1; **p = 0.05; ***p = 0.01

**Table 6:** The predictors of the multiple regression of minerals concentration in maternal serum (β-coefficient)

Amniotic fluid	Mg	Co	Cu	Cd	Se
Maternal age	− 15.57	0.00036	− 6.49	− 0.00025	0.60**
Maternal/newborn body weight	− 8.78	− 0.0025	26.40***	0.0012	0.33
Parity	− 272.84	0.0202	48.81	0.0025	1.62

*p = 0.1; **p = 0.05; ***p = 0.01

## Discussion

The concentration of essential and toxic elements in pregnant women and the fetal organism is affected by dynamic physiological changes in pregnant women as well as many factors such as maternal age, week of gestation, nutritional status and living environment. In this study, we assessed essential and toxic elements and established the reference values of 18 elements in AF and MS collected concurrently during delivery from healthy singleton pregnancies. We demonstrated that the concentration of the essential and toxic elements was lower in the AF than in the MS. Unexpectedly, the concentration of Pb was markedly higher in the AF than in the MS. To the best of our knowledge, this is the first study on healthy pregnant women to demonstrate these results.

The range of concentrations of some elements in the AF and MS in this study are in agreement with values investigated in earlier literature [8–11, 13, 14], although higher or lower levels of some specifications [8, 10, 11, 15–17] were reported. When compared with Nigerian women at the same week of gestation, the results for Cu, Zn and Fe in the serum of Polish women were markedly higher [5]. However, the level of toxic elements analyzed in the present study was higher in the AF of Polish women than in Italian women [10]. As regards to differences in the levels of trace elements observed in many similar studies, possible reasons include the week of gestation, maternal age, eating habits, health status and racial differences. The results presented herein were compared with values from biological material collected in the third trimester of pregnancy (28–42 weeks of gestation) because there are very few studies that focus on the results at the time of giving birth. To compare these results with our previous study, we observed a higher concentration of elements such as Mg, Sr, Cd, Ba, V, U and Cr in the AF of women during delivery than in women in the second trimester of pregnancy (16–26 weeks of gestation) [6]. The level of Cu and As in AF were lower in early pregnancy than in this study [6].

A lower quantity of elements in the AF than in MS was observed by other authors during the second trimester of gestation [7, 18]. Moreover, Silberstein et al. [7] found a significant correlation (*R* = 0.99) between the level of Fe, Zn, Co, Sr and B in AF and the serum of women at 16–21 weeks of pregnancy, although we have not confirmed it—neither in this nor our previous studies.

In this research, we found that the mean concentration of the most analyzed elements in the AF was approximately two times lower than in the MS. The lowest differences between the level in AF and MS were observed for Sr and V and the highest for Se and Cu. The concentration of Se in the MS was in the normal range for non-pregnant women [19] and was approximately 14 times higher than in the AF. However, the level of Cu in the serum of pregnant women during the third trimester was markedly higher than that reported in the literature for adult non-pregnant women [19]. Interestingly, the concentration of this microelement was 31 times lower in the AF than in the serum. In accordance with other data, our previous study [4] showed that Cu concentration in the blood of pregnant women was significantly higher than in non-pregnant women and increased during the course of the pregnancy [4, 15, 17, 20–22]. It is suggested that the Cu level is related to the increase in blood estrogen levels, which mobilizes Cu stores from tissues. Furthermore, pregnancy increases the maternal need for enzymes containing Cu and this leads to an increase in Cu absorption. Another potential reason may be decreased biliary Cu excretion induced by hormonal changes in pregnancy [8, 21].

The average Mg concentration in the serum was slightly lower than in non-pregnant women [19, 22]. It was confirmed that blood levels of Mg in pregnant women decreases as the pregnancy progresses [6, 15, 23]. Mg plays an important role in development during pregnancy and is utilized both for building the fetal body and metabolism in the fetus.

The concentrations of other essential minerals in the serum (Ca, Zn, Cr, Fe and Mn) were consistent with those reported in the literature for the normal adult population [19, 24]. This might indicate that the third trimester does not significantly affect the concentration of these elements in the serum of pregnant women, as seen from the samples collected at delivery.

Many maternal and fetal factors may affect mineral concentrations in the AF and MS [1]. In our previous study, we found that the body weight of pregnant women during the second trimester was negatively correlated with Mg concentrations in the AF [6] and this association has been confirmed in the present study. Generally, obesity is associated with micronutrient deficiency, and it was shown that hypomagnesemia frequently occurs in obese women [25]. Low Mg levels in AF are associated with pregnancy complications such as preeclampsia or gestational diabetes, and overweight and obese women are at higher risk of developing these diseases [26, 27]. In pregnant women, the BMI is usually inversely correlated with serum Cu level, although a positive correlation was observed between Cu concentration in the AF and fetal development [6]. However, Ugwuja et al. [5] found a negative correlation between the plasma Cu level and the newborn’s head circumference. In this study, we observed a positive association between the Cu level and the MBM/NBM ratio, which may be related to the increase in the mother’s Cu content during pregnancy [8, 20, 21, 23].

In pregnant women, an increased level of Se was observed, and the older the pregnant woman, the higher the level of this microelement [8]. The results of regression analysis obtained in this study partly confirmed this association.

Furthermore, in this study, we found that Pb content in the AF is markedly higher than in MS, although the level of Pb in the AF is higher when compared with reports from other studies [10]. Liu et al. observed that the serum Pb concentration increased during pregnancy [23]. The high concentration of Pb in AF in our study may be partly explained by maternal Pb exposure during pregnancy, which is readily transferred to the fetus through the placenta. Moreover, a protective mechanism can prevent the accumulation of Pb in the fetus by excreting Pb into the AF.

## Study Limitations

This study has some limitations. Firstly, we analyzed only certain essential and toxic elements in the AF and MS from pregnant women. Secondly, in this study, we included only women at delivery because they were at the end of the third trimester of pregnancy. Furthermore, in the present study, we did not determine nutritional factors and environmental factors that may affect the concentrations of elements in the analyzed samples. Finally, we carried out research among a limited group of healthy women; however, in the future, we plan to expand the diversity of the study population and include groups of women with pathological conditions such as preeclampsia, intrauterine growth restriction, spontaneous preterm birth, gestational diabetes, and fetal macrosomia as well as cases with fetal congenital defects, genetic disorders, and chromosomal abnormalities.

## Conclusions

The reference ranges of 18 essential and toxic elements in the AF and MS at delivery were established. The MBM/NBM ratio may determine the concentration of Mg and Cu in the AF and MS. Maternal age is a predictor of serum Se concentration during the third trimester of gestation.

## Electronic supplementary material

ESM 1 (PDF 67 kb)

ESM 2 (PDF 57 kb)

ESM 3 (PDF 74 kb)

ESM 4 (PDF 59 kb)

ESM 5 (DOCX 12 kb)
