# Supplementary figures and images for: Evaluation of Essential and Toxic Elements in Amniotic Fluid and Maternal Serum at Birth
Source: Biol Trace Elem Res. 2018 Aug 10;189(1):45–54. doi: 10.1007/s12011-018-1471-2 (PMC6443612; doi:10.1007/s12011-018-1471-2)

**Mg\_S**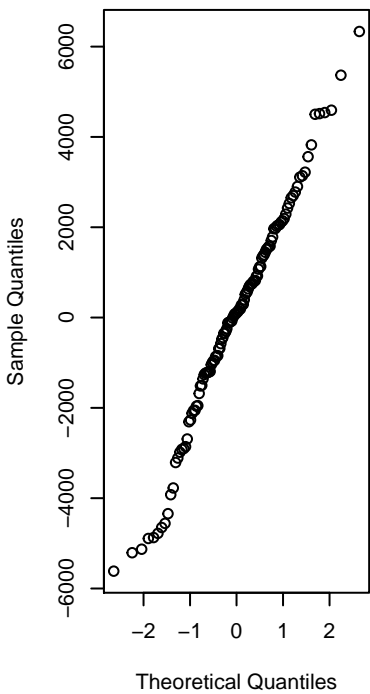**Co\_S**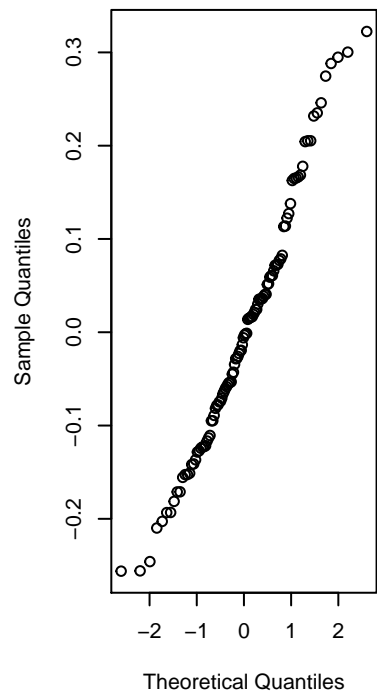**Cu\_S**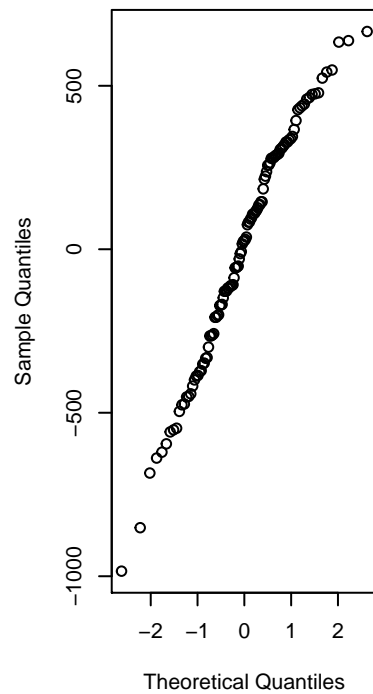**Zn\_S**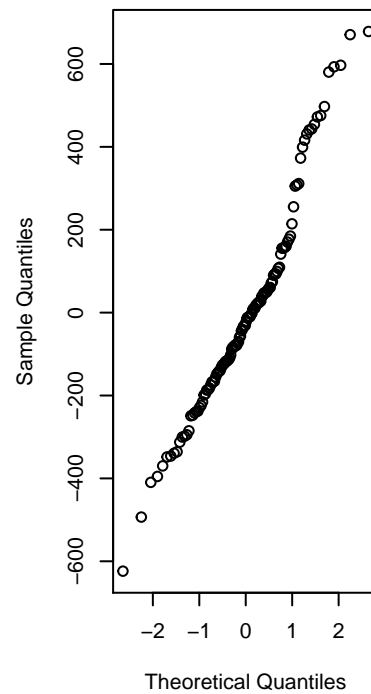**Sr\_S**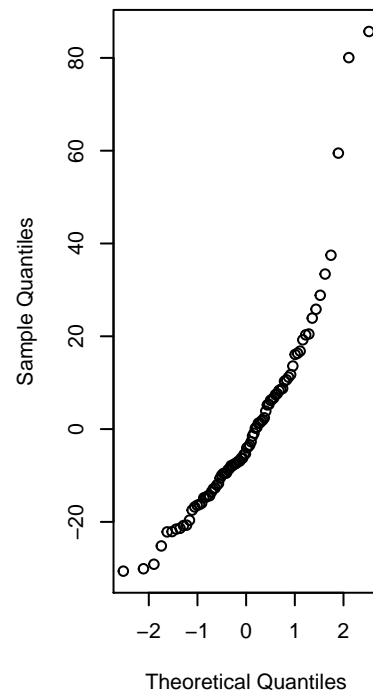**Cd\_S**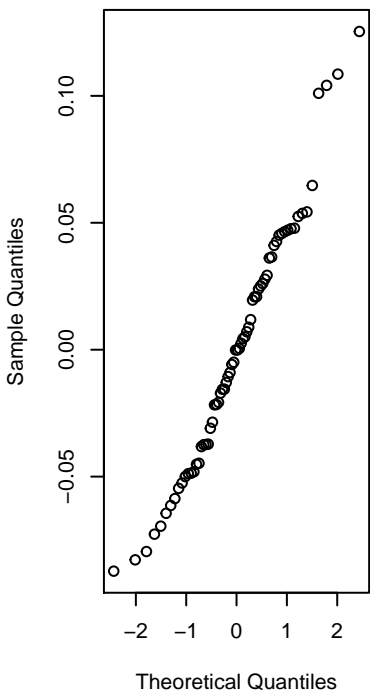**Ba\_S**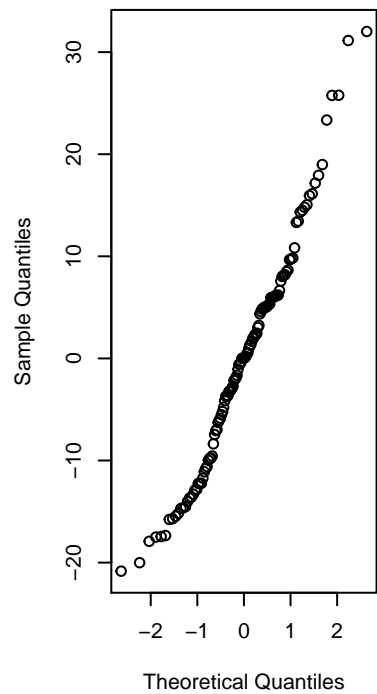**Pb\_S**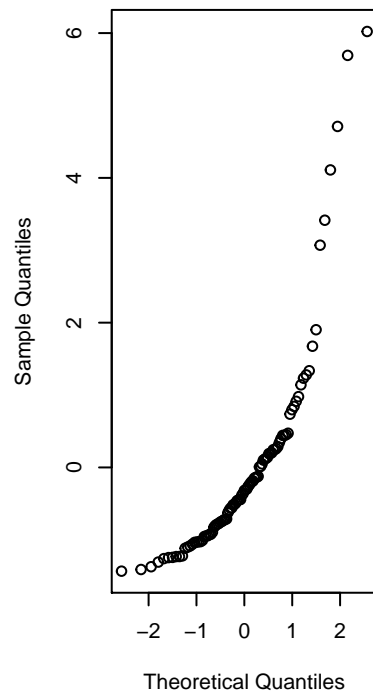**U\_S**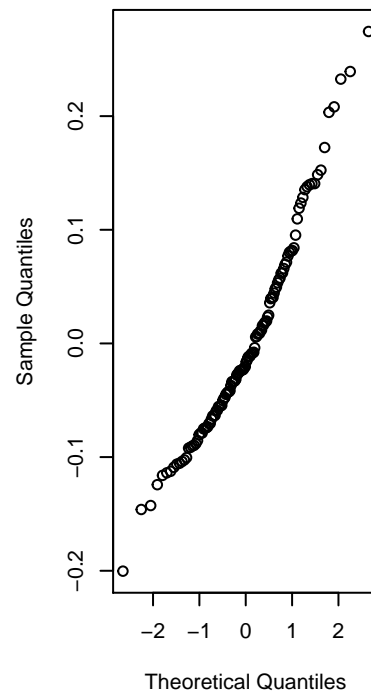**Ca\_S**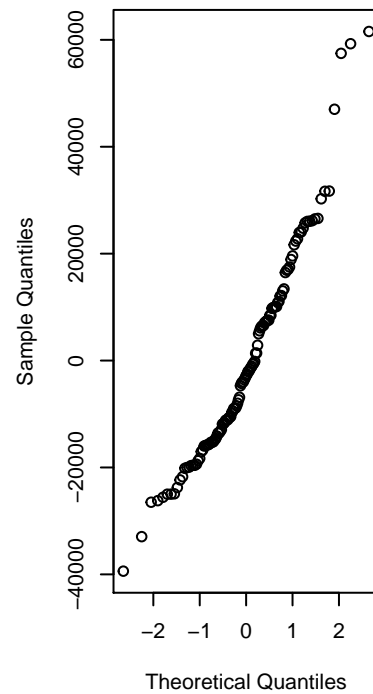

Supplement: Supplementary file 1 — (PDF 67 kb) [file 12011_2018_1471_MOESM1_ESM.pdf]

**Cr\_S**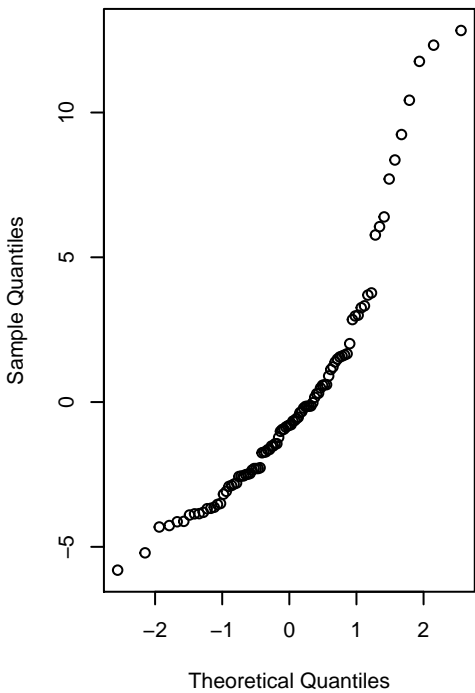**Al\_S**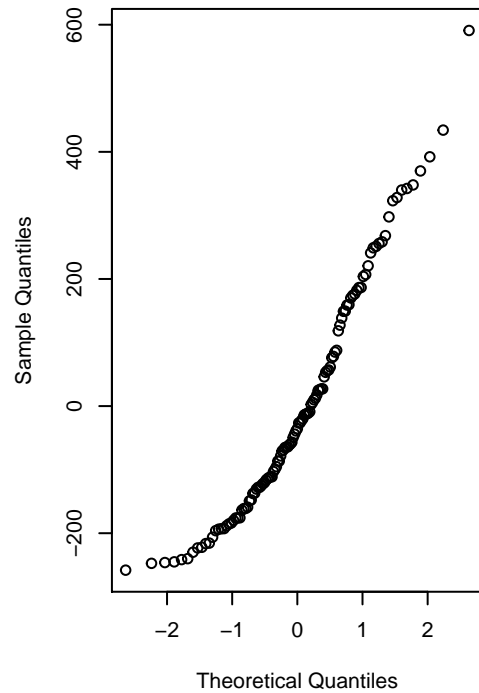**Mn\_S**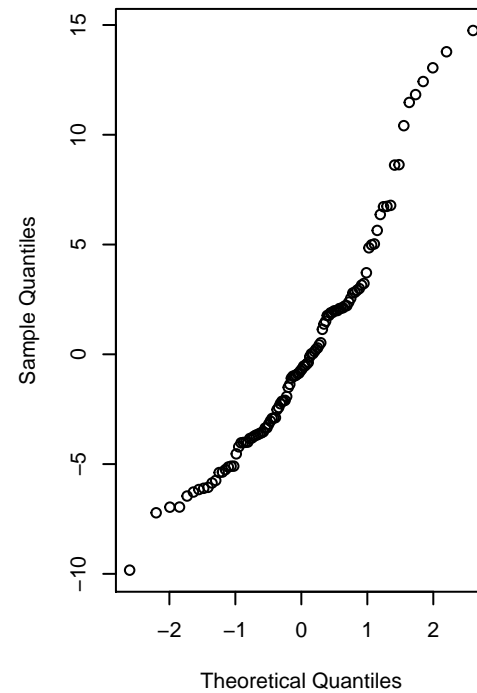**V\_S**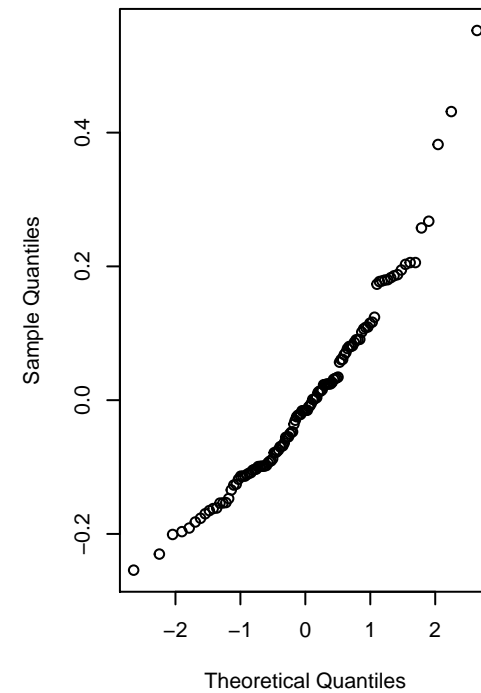**Fe\_S**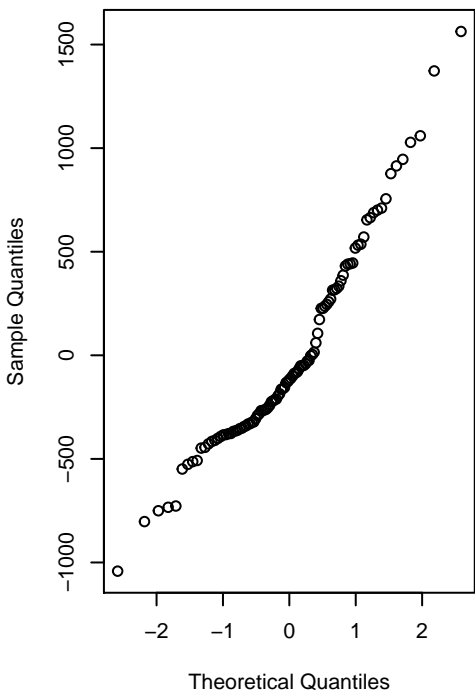**As\_S**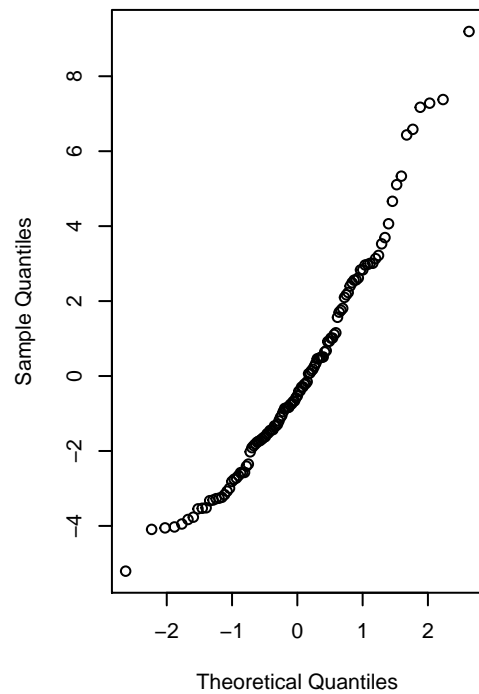**Se\_S**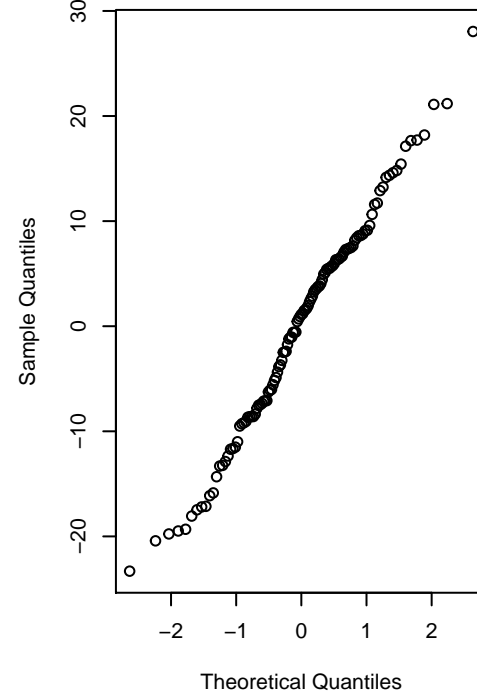**Sb\_S**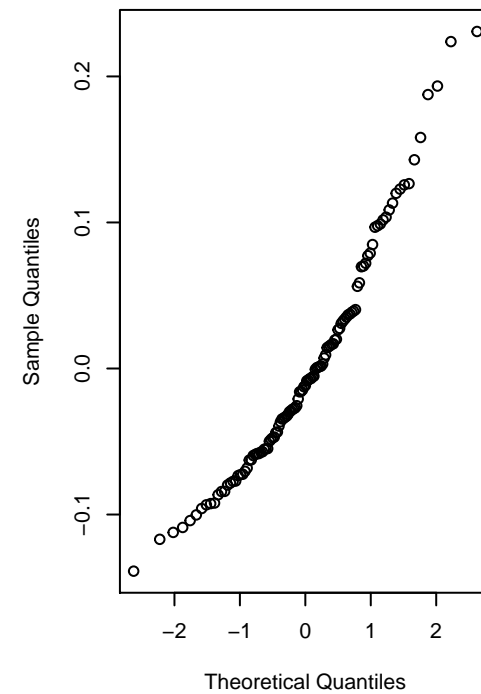

Supplement: Supplementary file 2 — (PDF 57 kb) [file 12011_2018_1471_MOESM2_ESM.pdf]

**Cr\_AF**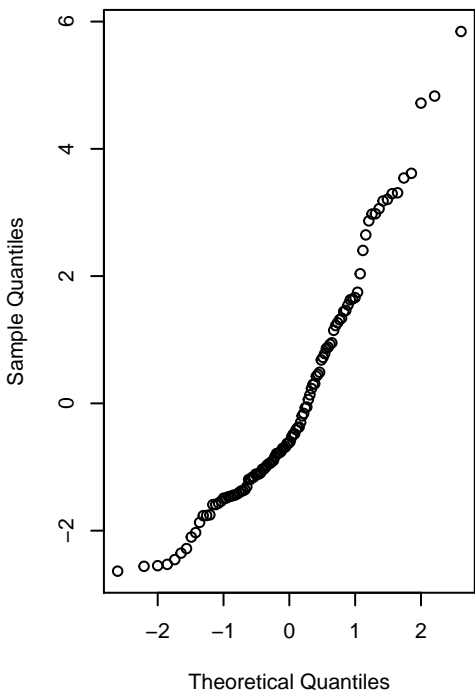**Al\_AF**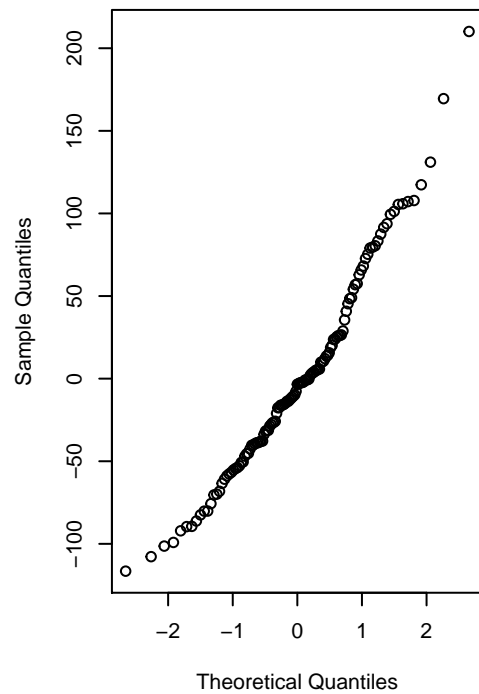**Mn\_AF**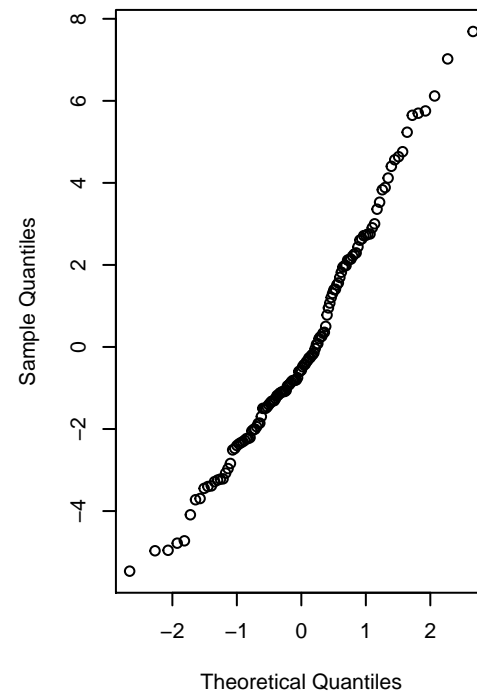**V\_AF**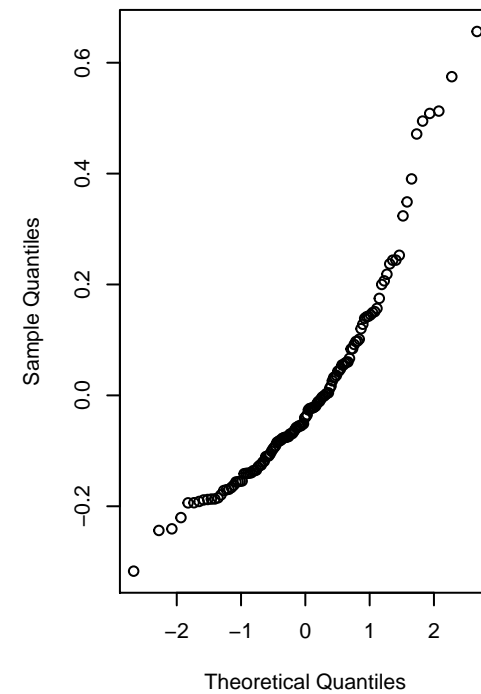**Fe\_AF**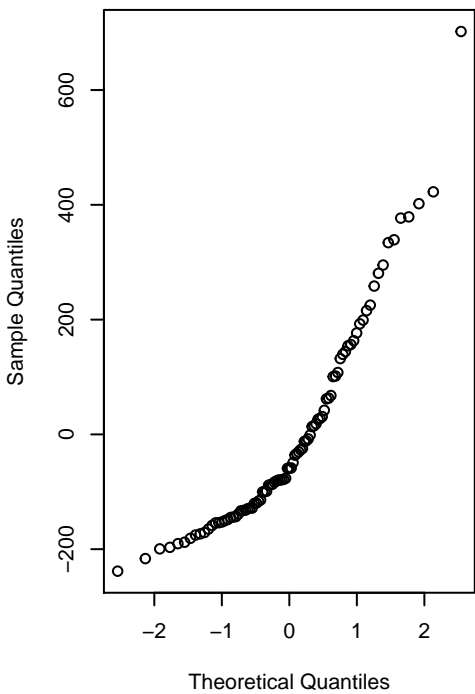**As\_AF**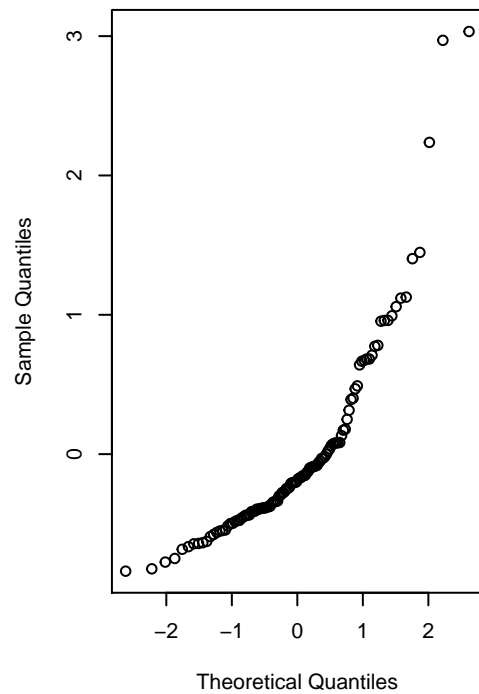**Se\_AF**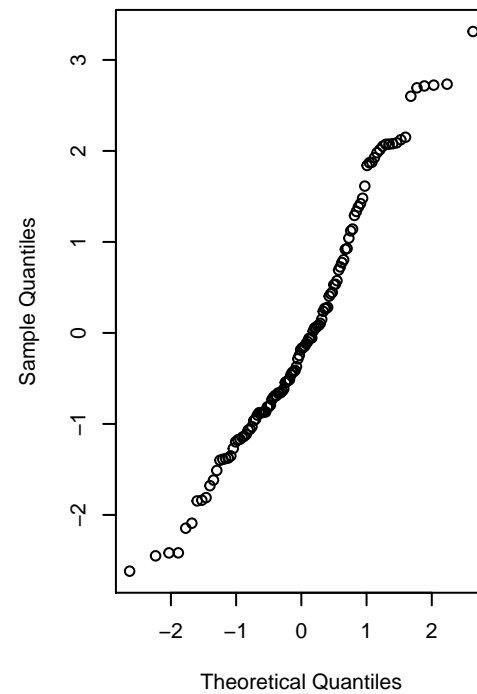**Sb\_AF**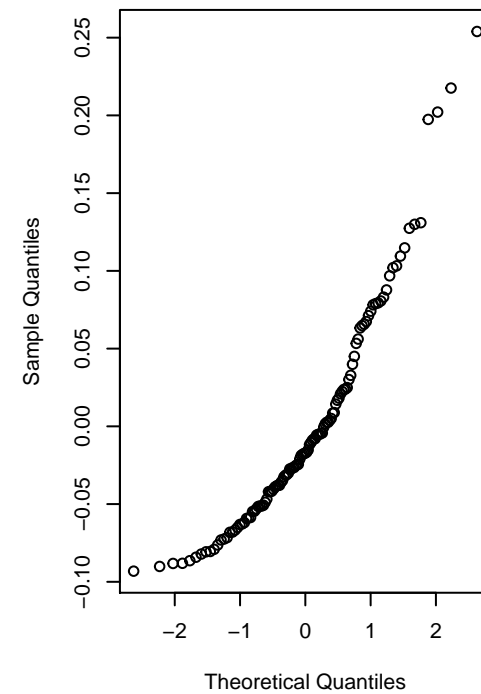

Supplement: Supplementary file 4 — (PDF 59 kb) [file 12011_2018_1471_MOESM4_ESM.pdf]
