# Supplementary material for: Evaluation of Essential and Toxic Elements in Amniotic Fluid and Maternal Serum at Birth
Source: Biol Trace Elem Res. 2018 Aug 10;189(1):45–54. doi: 10.1007/s12011-018-1471-2 (PMC6443612; doi:10.1007/s12011-018-1471-2)
Supplement: Supplementary file 3 — (PDF 74 kb) [file 12011_2018_1471_MOESM3_ESM.pdf]

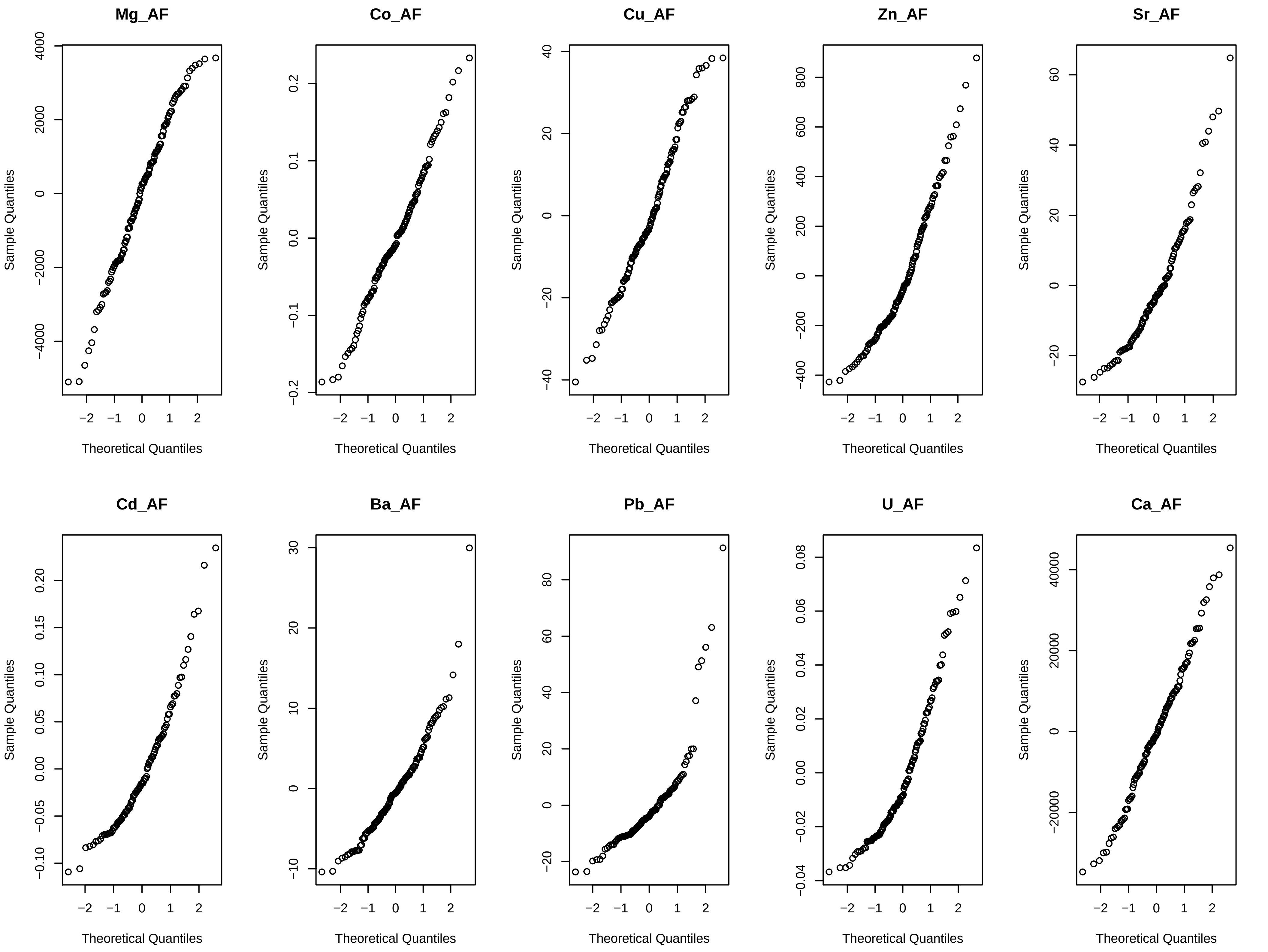

Mg_AF
Sample Quantiles
4000
2000
0
−2000
−4000
Theoretical Quantiles
−2
−1
0
1
2
Co_AF
0.2
0.1
0.0
−0.1
−0.2
Cu_AF
40
20
0
−20
−40
Zn_AF
800
600
400
200
0
−200
−400
Sr_AF
60
40
20
0
−20
Cd_AF
0.20
0.15
0.10
0.05
0.00
−0.05
−0.10
Ba_AF
30
20
10
0
−10
Pb_AF
80
60
40
20
0
−20
U_AF
0.08
0.06
0.04
0.02
0.00
−0.02
−0.04
Ca_AF
40000
20000
0
−20000
