# Supplementary material for: Evaluation of Essential and Toxic Elements in Amniotic Fluid and Maternal Serum at Birth
Source: Biol Trace Elem Res. 2018 Aug 10;189(1):45–54. doi: 10.1007/s12011-018-1471-2 (PMC6443612; doi:10.1007/s12011-018-1471-2)
Supplement: Supplementary file 5 — (DOCX 12 kb) [file 12011_2018_1471_MOESM5_ESM.docx]

Fig. 2ab. (supplementary) The normality of the residuals in maternal serum.

Fig. 3ab. (supplementary) The normality of the residuals in amniotic fluid.
